# Integrating optical imaging techniques for a novel approach to evaluate Siberian wild rye seed maturity

**DOI:** 10.3389/fpls.2023.1170947

**Published:** 2023-04-20

**Authors:** Zhicheng Jia, Chengming Ou, Shoujiang Sun, Juan Wang, Jingyu Liu, Ming Sun, Wen Ma, Manli Li, Shangang Jia, Peisheng Mao

**Affiliations:** College of Grassland Science and Technology, China Agricultural University, Beijing, China

**Keywords:** Siberian wild rye seed, feature filtering, machine learning, integrating optical imaging, multispectral imaging, autofluorescence imaging, model updating

## Abstract

Advances in optical imaging technology using rapid and non-destructive methods have led to improvements in the efficiency of seed quality detection. Accurately timing the harvest is crucial for maximizing the yield of higher-quality Siberian wild rye seeds by minimizing excessive shattering during harvesting. This research applied integrated optical imaging techniques and machine learning algorithms to develop different models for classifying Siberian wild rye seeds based on different maturity stages and grain positions. The multi-source fusion of morphological, multispectral, and autofluorescence data provided more comprehensive information but also increases the performance requirements of the equipment. Therefore, we employed three filtering algorithms, namely minimal joint mutual information maximization (JMIM), information gain, and Gini impurity, and set up two control methods (feature union and no-filtering) to assess the impact of retaining only 20% of the features on the model performance. Both JMIM and information gain revealed autofluorescence and morphological features (CIELab A, CIELab B, hue and saturation), with these two filtering algorithms showing shorter run times. Furthermore, a strong correlation was observed between shoot length and morphological and autofluorescence spectral features. Machine learning models based on linear discriminant analysis (LDA), random forests (RF) and support vector machines (SVM) showed high performance (>0.78 accuracies) in classifying seeds at different maturity stages. Furthermore, it was found that there was considerable variation in the different grain positions at the maturity stage, and the K-means approach was used to improve the model performance by 5.8%-9.24%. In conclusion, our study demonstrated that feature filtering algorithms combined with machine learning algorithms offer high performance and low cost in identifying seed maturity stages and that the application of k-means techniques for inconsistent maturity improves classification accuracy. Therefore, this technique could be employed classification of seed maturity and superior physiological quality for Siberian wild rye seeds.

## 1 Introduction

The genus *Elymus* (L.), a member of the grass family (Poaceae), is the most widespread genus in the northern hemisphere, with approximately 150 species. Some species of *Elymus* exhibit remarkable resilience to biotic and abiotic stresses, including drought, cold, and disease. Siberian wild rye (*E. sibiricus* L.) is a notable representative with a wide distribution in the northern regions of Eurasia. Due to its broad adaptability, high cold tolerance, high nutritional value, palatability, and ease of cultivation, Siberian Wild rye has been widely used for grassland restoration and fodder production (Klebesadel, 1969; You et al., 2011; Xie et al., 2020). However, its seed yield remains relatively low, with only about 20% of its potential production being harvested. Research suggests that excessive shattering is a major factor contributing to this low seed yield, which has been confirmed by various studies (Yu et al., 2011; Zhao et al., 2012; Han et al., 2013; Zhao et al., 2015).

The traditional method of determining the optimum time to harvest Siberian wild rye seed relied on the subjective judgment of experienced farmers, which lacked objectivity and often resulted in sub-optimal yields. Meanwhile, to assess the physiological potential of Siberian wild rye seed at different maturity stages, conventional methods such as germination tests and physiological experiments were employed, which were both laborious and destructive (Rahman and Cho, 2016). With the rise of the modern seed industry and the growing demand for smart agriculture, these conventional methods have become increasingly inadequate to meet the needs of the industry. There is therefore an urgent need to develop a rapid, non-destructive, high-throughput method for identifying and classifying the maturity of Siberian wild rye seeds. Such a method would enable the modern seed industry to maximize yield and quality and meet the growing demand for high-quality seeds (Feng et al., 2019).

Biological imaging techniques, including X-ray, hyperspectral, multispectral, and autofluorescence optical technologies, have brought about significant changes in agricultural production and food quality. The multispectral imaging technology was a non-destructive technique that combined computer vision and spectroscopy to provide information on physical attributes such as texture, color, shape, size, and chemical composition (ElMasry et al., 2019; França-Silva et al., 2020). The main principle of the technique was based on the detection of different specific wavelengths produced by the varying physical structures and chemical compositions of objects. For example, multispectral imaging has been successfully used to identify variety genuineness and seeds quality, such as alfalfa (*Medicago sativa* L.) seeds (Yang et al., 2020; Jia et al., 2022), manioca (*Jatropha curcas* L.) seeds (Pinheiro et al., 2020), and spinach (*Spinacia oleracea* L.) seeds (Deleuran et al., 2013). The imaging technology allowed a better understanding of the seed maturation process and provided a research basis for the development of rapid, non-destructive, and high-throughput detection methods. The autofluorescence spectral imaging technique was based on the detection of fluorescence group signals in seeds that changed during maturation (Teixeira et al., 2016; Lima et al., 2017). With the advancement of optical imaging devices, integrated optical imaging devices had started to be applied in various fields such as food, medicine, and agriculture (Galletti et al., 2020; Wang et al., 2021).

In recent years, advanced imaging devices that integrate multi-optical components (multispectral, autofluorescence, hyperspectral, and RGB) can provide more comprehensive information and thus improve detection accuracy, but increase the dimensionality of the data and place higher demands on the computing equipment. The successive projection algorithm (SPA), a linear algorithm, is widely used to select important spectral bands in hyperspectral images (Tu et al., 2022). However, there are challenges with multi-source fused non-linear datasets, and to overcome this challenge, it was imperative to explore alternative feature selection methods that could reduce the dimensionality of the data, as high-dimensional data could lead to computational inefficiencies and prolong the training time of machine learning (ML) algorithms. For example, feature filtering algorithms such as minimal joint mutual information maximization (JMIM) (Kursa, 2018), information gain (Zawadzki and Kosinski, 2019), and Gini impurity (Bommert et al., 2020) have been shown to effectively reduce the dimensionality of high-dimensional data. However, current researches were mainly on individual datasets (França-Silva et al., 2020; Fu et al., 2023), while little research has been reported on the use of feature filtering algorithms on multi-source fused data.

## 2 Materials and methods

### 2.1 Sample materials

Samples were collected from Yuershan Ranch in Chengde City, Hebei Province, China. 100 spikelets were randomly selected from the field and stored in liquid nitrogen, and then transported to the laboratory for further analysis. These samples were stored in a refrigerator at minus 20 degrees Celsius for the determination of physiological indices. Additionally, 200 spikelets were air-dried and stored for germination testing and multispectral image acquiring. To ensure consistency among the samples, 4 - 6 spikelets were selected from the middle of the spike, and the 1st and 2nd seeds at the base of the spikelets were separated from the rest of the seeds and labeled as superior grain (SG) and inferior grain (IG) respectively. Samples were collected at the milk-ripe stage (MRS) (July 27), dough stage (DS) (August 7), and full-ripe stage (FRS) (August 13), and the dry weight, fresh weight, and moisture content are shown in **Table 1**.

**Table 1:** The information of seed dry weight, fresh weight and water content at MRS, DS, and FRS.

Stages	Grain position	Fresh weight (mg/grain)	Dry weight (mg/grain)	Water content (%)
MRS	SG	8.06 ± 0.07a	3.85 ± 0.04b	52.20 ± 0.26b
MRS	IG	6.79 ± 0.02b	3.06 ± 0.04c	54.91 ± 0.55a
DS	SG	6.63 ± 0.06b	4.38 ± 0.04a	33.95 ± 0.83c
DS	IG	6.27 ± 0.02c	4.33 ± 0.06a	30.89 ± 0.95d
FRS	SG	5.29 ± 0.06d	4.38 ± 0.06a	17.32 ± 0.77e
FRS	IG	5.27 ± 0.08d	4.33 ± 0.04a	17.77 ± 0.45e

(± SD). Different lowercase letters indicated significant differences in Siberian wild rye seeds at different stages and grain positions at the P<0.05 level.

### 2.2 Germination test

A germination experiment was carried out with Siberian wild rye seeds of uniform size. The experiment was repeated four times with 8 hours of light and 16 hours of darkness, with a light intensity of 66% and a fluctuating temperature of 15/25°C. Initial and final germination counts were made after 5 and 12 days respectively, and shoot and root length measurements were taken at the final germination count. The number of seeds with radicles greater than 2 mm was recorded every 24 hours during the germination period. Finally, the germination percentage and the germination speed index were calculated.


(1) Germination percentage = (G10/N)×100%

(2) Germination speed index = ∑(n/t)

Where *G10* was the number of normal seedlings at the last count, *N* was the total number of experimental seeds, *n* was the number of seed germination per day, and *t* was the number of days per germination.

### 2.3 Autofluorescence and multispectral imaging

Multispectral images were acquired from seeds using a VideometerLab4™ device (Videometer A/S, Herlev, Denmark). The system incorporates a CCD chip with 19 wavelengths of high-powered light-emitting diodes (LEDs) arranged around the edge of the sphere at 365, 405, 430, 450, 470, 490, 515, 540, 570, 590, 630, 645, 660, 690, 780, 850, 880, 940, and 970 nm, ranging from ultraviolet (UV) to near-infrared (NIR), the led flashes continuously in a few seconds of scan time, producing monochrome images at 19 different wavelengths (2192 × 2192 pixels; 40 µm/pixel; 32 bits/pixel). And autofluorescence images were acquired by a mounted long-pass (LP) filter combined with different excitation wavelengths, which offers the following excitation-emission combinations: 365/400 nm, 365/500 nm, 405/500 nm, 430/500 nm, 450/500 nm, 630/700 nm, 645/700 nm, 660/700 nm.

### 2.4 Autofluorescence and multispectral image analysis

After acquiring the images, each seed was segmented from the background into a region of interest (ROI) using VideometerLab 3.14 software. All seeds were collected and added to a blob database, from which we extracted morphological features, multispectral features, and autofluorescence features of the seeds. A detailed description of the morphological features used in this study can be found in **Table S1**, while the multispectral features and autofluorescence features are described in section 2.3. The extracted multispectral and fluorescence features of individual ROI seeds represent the average reflected light intensity at each single wavelength calculated from all pixels in a single ROI image. In total, 42 features (cols) and 600 samples (rows) of data were used in this study. All three types of features for seeds were then collected in a matrix (X), associated with their corresponding stages and grain positions data (Y).

### 2.5 Data analysis

In this study, we conducted a comprehensive analysis of the morphological features, multispectral, and autofluorescence properties of Siberian wild rye seeds. To statistically evaluate differences among seed maturity stages and grain positions, we employed both Duncan’s test and Student’s t-test (*P*< 0.05). To reduce the number of features, we utilized three feature filtering methods: JMIM, information gain, and Gini impurity. We calculated the feature importance scores for all 42 features (**Table S2**), and based on these scores, we selected 20% as the threshold. This means that only 20% of the features were retained for further analysis. Furthermore, we included a features union approach (where filtering features of the three algorithms were fused using the union method) and a no-filtering group as control groups. Therefore, a total of five feature filtering methods were employed to analyze the data.

Principal component analysis (PCA), linear discriminant analysis (LDA), support vector machine (SVM), and random forest (RF) were applied in this study. PCA generally is used to reduce the dimensionality of the data as a mathematical technique by an orthogonal transformation of the initial data set into a new set of uncorrelated variables, the so-called principal components (PCs), where the first PC has the highest variance, the second PC has the second-highest variance, and so on. Thus, key information and potential data structure of high-dimensional data can be provided by PCs. LDA, a classical ML algorithm, calculates the optimal transformation (projection) by simultaneously minimizing the within-class distance and maximizing the between-class distance, resulting in maximum discrimination. SVM, a well-known kernel method, has been effectively used for multivariate function estimation or nonlinear classification by finding the optimal hyperplane to achieve segmentation of high-dimensional data (Cristianini and Shawe-Taylor, 2000).

**Figure 1:**
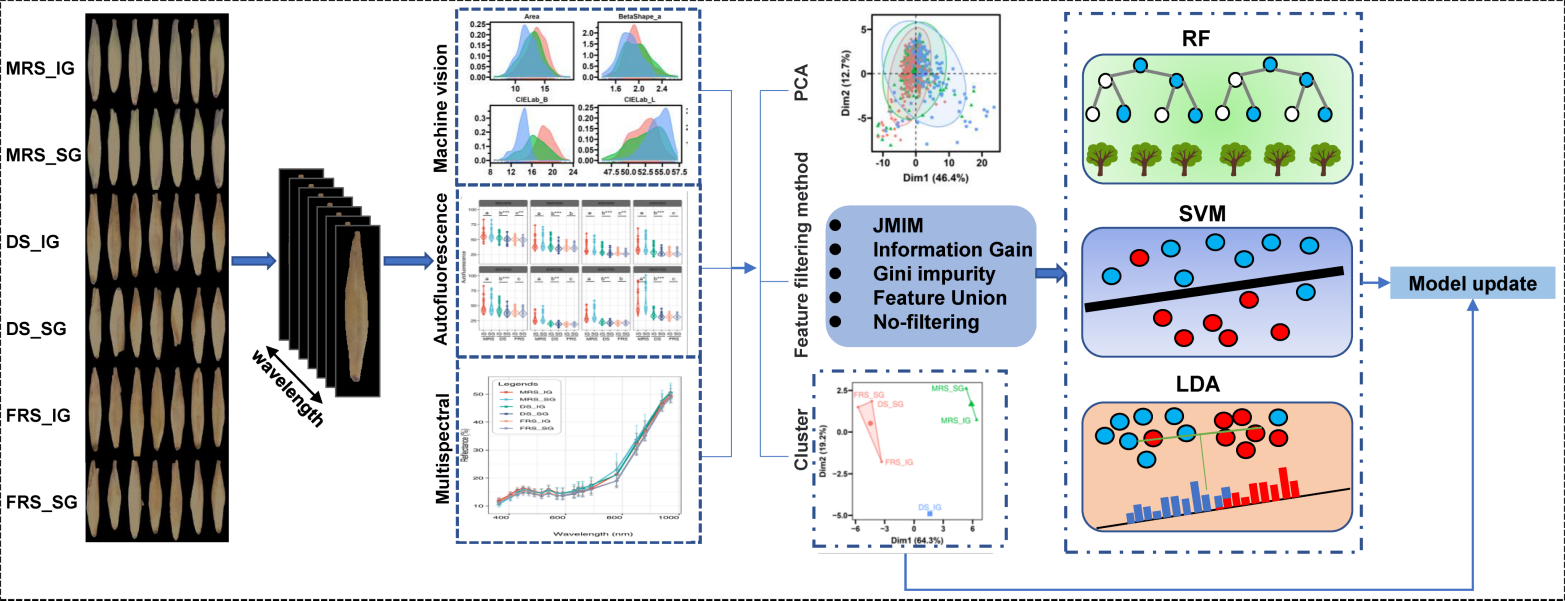
Technical route. RF, random forest; SVM, support vector machine; LDA, linear discriminant analysis. We obtained RGB images, multispectral images, and autofluorescence images of Siberian wild rye seeds at different maturity stages and grain positions using Vidoemeter equipment. Then, we segmented individual seeds from the background and extracted their morphological, multispectral, and autofluorescence features. Furthermore, we performed PCA and LDA exploratory analysis on the three types of features and their multi-source fusion data. We also selected three filtering algorithms, JMIM, information gain, and Gini impurity, and set two controls (feature fusion and non-filtered features) to filter the multi-source fusion data. Based on the filtered features, we built SVM, RF, and LDA models to differentiate different seed maturity stages. Additionally, we applied k-means clustering to reclassify seeds based on their maturity stages and grain positions, and updated the model to further improve its classification performance.

In this study, we evaluated model performance for multiclass classification tasks using accuracy, area under curve (AUC), and Brier score (Brier, 1950). We implemented the LDA, RF, and SVM algorithms using the ‘mlr3verse’ R package (Lang and Schratz, 2021) in R 4.1 software. The PCA and K-means were implemented by ‘FactoMineR’ and ‘cluster’ R packages, respectively (Lê et al., 2008; Maechler et al., 2012). Additionally, we optimized the hyperparameters of the SVM and RF models using 5-fold cross-validation and the random search method. The optimized parameters were listed in **Table S3**, while the other parameters were set to their default values. The technology route for this study was illustrated in **Figure 1**.

## 3 Result

### 3.1 Effect of maturity and grain position of spikes on seed germination

**Figure 2:**
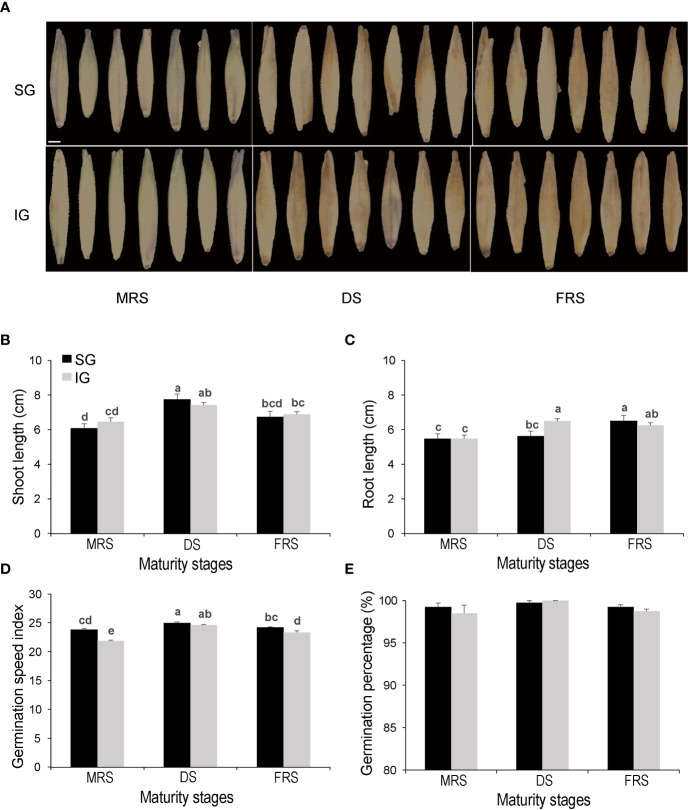
Germination and vigor tests, sorting of Siberian wild rye seeds at different developmental stages, and grain positions. **(A)** Seed images. **(B)** Shoot length. **(C)** Root length. **(D)** Germination speed index. **(E)** Germination percentage (%). Different lowercase letters indicate significant differences at different stages and grain positions at the P<0.05 level.

The results indicate that there were differences in the quality of Siberian wild rye seeds at different maturity stages and grain positions. The analysis revealed that seeds from the MRS exhibited a greener color compared to those from the DS and FRS. However, there was no observable difference in the appearance of seeds from the DS and FRS. Moreover, there was no discernible distinction between seeds from different grain positions within the same maturity stage in terms of appearance (**Figure 2A**). Shoot length initially increased and then decreased as maturity increased, with the highest values observed in the DS-SG seeds, which were significantly higher than those of the other maturity stage seeds (*P*<0.05) (**Figure 2B**). Meanwhile, a gradual increase in root length was observed for both SG and IG samples as the maturity stage progressed (**Figure 2C**). Furthermore, the germination speed index of the SG was significantly higher than that of the IG seeds during the MRS and FRS (*P*<0.05) (**Figure 2D**). However, there were no significant differences in germination percentage among different maturity stages and grain positions, with germination percentages of all seeds consistently close to 100% (**Figure 2E**).

### 3.2 Morphological, multispectral, and autofluorescence data analysis

**Figure 3:**
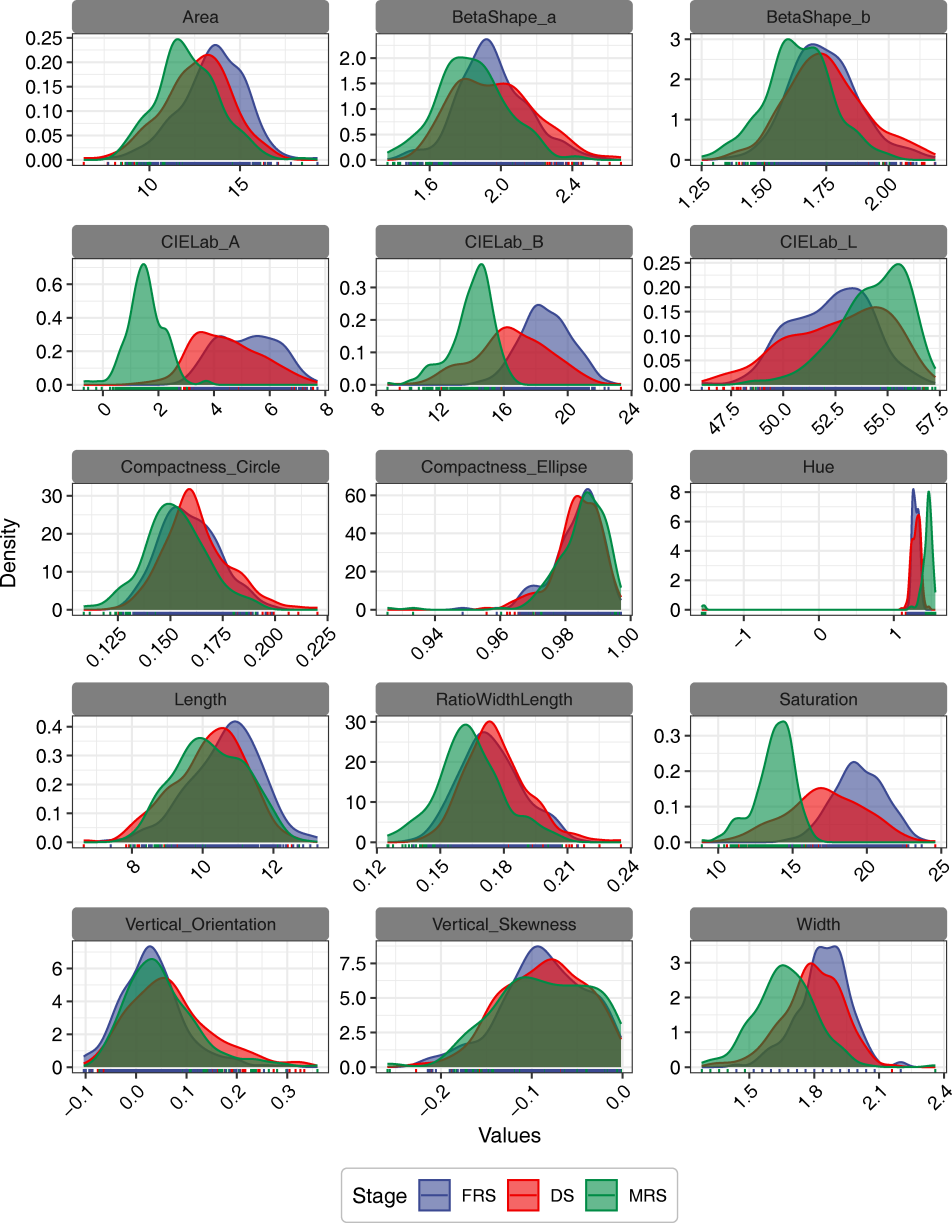
The probability density distributions of morphological features of Siberian wild rye seeds for different stages and grain positions.

Fourteen morphological features were extracted from RGB images of seeds from three developmental stages and two grain positions. The probability density distribution trends of MRS seeds in saturation, CIELab A, and CIELab B were significantly different from those of seeds in the other two maturity stages (**Figure 3**). Our statistical analysis of the morphological features showed that the values of these three features were significantly lower than those of the other seeds, with the lowest value observed in IG seeds (*P*<0.05) (**Table S4**). On the other hand, the probability density distributions of seeds in the three maturity stages were not clearly differentiated for other morphological features.

**Figure 4:**
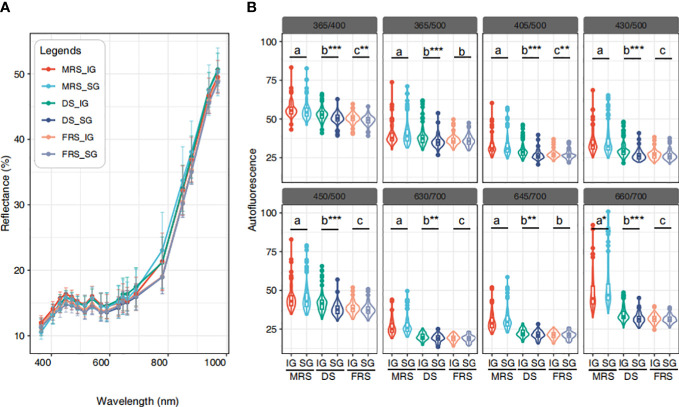
Spectral information of Siberian wild rye seeds. **(A)** Average multispectral reflectance of 19 wavelengths. **(B)** 8 autofluorescence wavelengths. The use of different colours was used to indicate differences in grain position at different maturity stages. The Duncan test was used to determine the significance of differences among maturity stages of Siberian wild rye seeds at the *P*<0.05 level, as indicated by the use of lowercase letters. In addition, Student’s t-test was used to determine the significance of differences between grain positions within the same maturity period, as indicated by the use of symbols ‘*’, ‘**’, ‘***’ or the absence of such symbols, respectively, denoting significance at *P*<0.05, *P*<0.01, *P*<0.001, and non-significance at *P*>0.05.

Overall, the mean multispectral reflectance of seeds from different maturity stages and grain positions exhibited similar trends. We observed that the average reflectance increased as the wavelength increased (**Figure 4A**). Specifically, in the spectral range of 365 to 570 nm, seeds from the MRS-IG exhibited the highest reflectance, while seeds from the FRS-SG exhibited the lowest reflectance. In the NIR range (780 - 970 nm), we found that the multispectral reflectance of seeds from the MRS-SG was significantly higher compared to the other seed classes (*P*<0.05) (**Table S5**).

We further extracted eight autofluorescence spectra wavelengths, and the results showed that the autofluorescence spectra decreased progressively as the maturity of Siberian wild rye seeds increased. In particular, the average autofluorescence of SG was lower (or similar) than that of IG in the same maturity stage. Furthermore, the autofluorescence of MRS seeds was the highest in all eight autofluorescence bands. The MRS seeds were effectively detected at 365/400 nm, 365/500 nm, 405/500 nm, 430/500 nm, 450/500 nm, 630/700 nm, 645/700 nm, and 660/700 nm excitation-emission combinations, where 365/400 nm, 405/500nm, 430/500 nm, 450/500 nm, and 660/700 nm provided finer classifications for three maturity stage seeds (**Figure 4B**). For the SG and IG classifications at different maturity stages, the SG and IG seeds of DS could be distinguished by all autofluorescence bands. However, only 660/700 nm provided a stronger separation of IG and SG in MRS, and 365/400 nm in FRS allowed a clear classification of IG and SG seeds.

**Figure 5:**
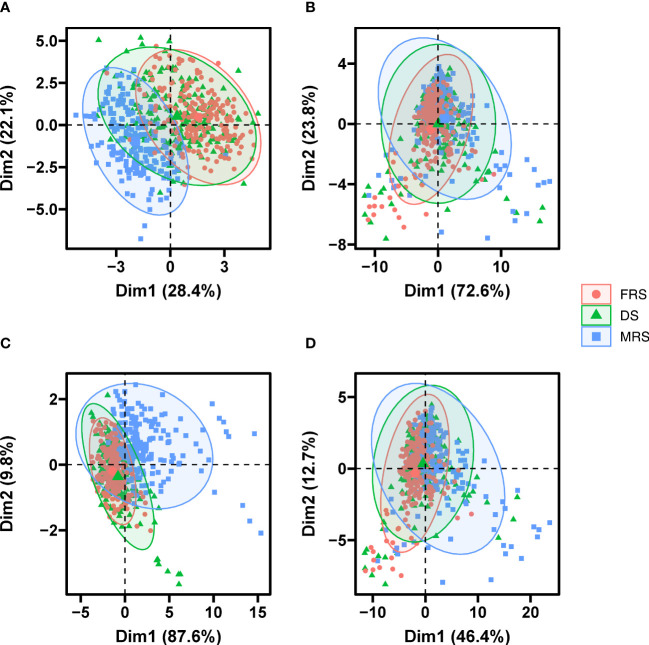
PCA score plot of seeds of different maturity stages and grain positions based morphological data **(A)**, multispectral data **(B)**, autofluorescence data **(C)**, and multi-source fusion data **(D)**.

The results of the PCA analysis showed that the first two PCs for morphological features accounted for 50.5% of the total variation among developmental stages, with 28.4% for PC1 and 22.1% for PC2 (**Figure 5A**). In addition, for multispectral features, the first two PCs explained 72.6% and 23.8% of the variation, respectively (**Figure 5B**). The autofluorescence spectral features also showed a similar trend, with the first two PCs explaining 87.6% and 9.8% of the variance, respectively (**Figure 5C**). Furthermore, when all three features were considered, the first two PCs (46.4% for PC1 and 12.7% for PC2) accounted for a total of 59.1% of the original variance (**Figure 5D**). Despite the high variation explained by the first two PCs based on autofluorescence spectral features, no significant differences were observed between Siberian wild rye seeds at the three maturity stages.

**Figure 6:**
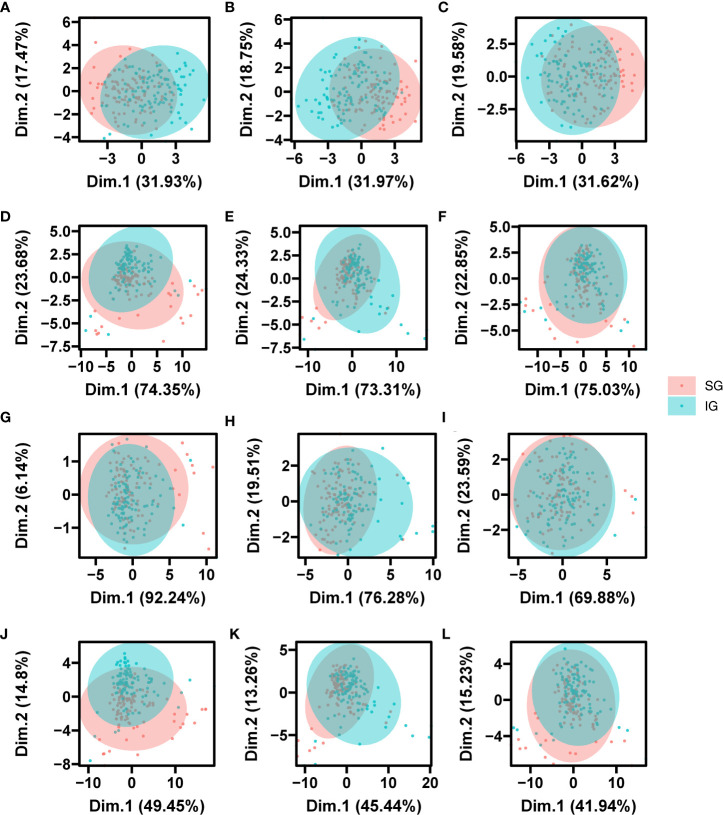
PCA score plot of grain position of seed based on the morphological data, the autofluorescence data, the multispectral data and multi-source fusion data. Figure **(A–C)** was morphological data, Figures **(D–F)** was autofluorescence data, **(G–I)** was multispectral data, and **(J–L)** was multi-source fusion data. Figures **(A, D, G, J)** were MRS; Figures **(B, E, H, K)** were DS; Figures **(C, F, I, L)** were FRS.

We performed PCA on seed morphological, multispectral, and autofluorescence features for different grain positions at the same maturity stage. The results showed that the first two PCs for morphological features at the three developmental stages accounted for 49.33-51.2% of the total variation between grain positions, with 31.62-31.97% for PC1 and 17.47-19.58% for PC2, respectively (**Figures 6A–C**). For multispectral features, the first two PCs explained approximately 73% and 23% of the variance, respectively (**Figures 6D–F**). For autofluorescence spectral features, PC1 explained 92.4% of the variation during MRS, while PC1 explained 73.31-75.03% of the variation at different grain positions for DS and FRS (**Figures 6G–I**). Furthermore, based on the combination of the three characters, the first two PCs (46.4% for PC1 and 12.7% for PC2) together explained about 48% of the original variation (**Figures 6J–L**). In summary, the different grain position seeds in the four datasets could not be completely distinguished; however, the different grain position seeds in MRS and DS did not completely overlap with each other compared to FRS.

**Figure 7:**
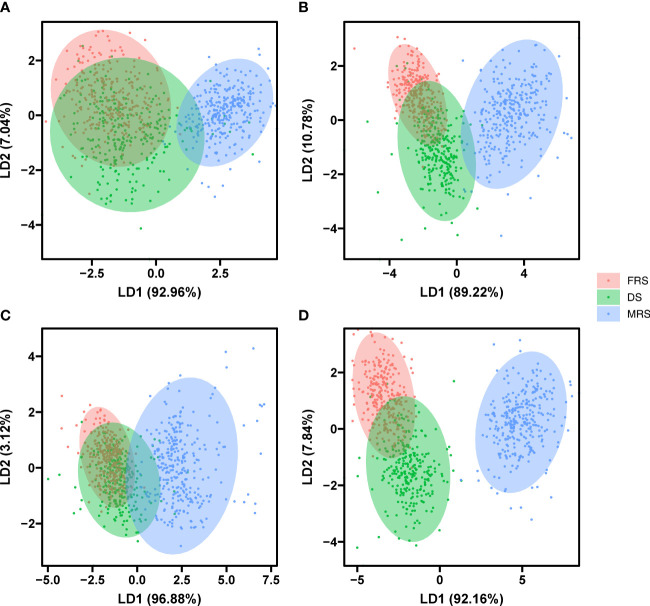
Two-dimensional biplot of LDA scores distinguishing seeds of three stage Siberian wildrye seeds based on morphological data **(A)**, multispectral data **(B)**, autofluorescence data **(C)**, and multi-source fusion data **(D)**.

The LDA results indicated that the first two LDs explained 92.96% and 7.04% of the variance in morphological traits, respectively. However, the limited variation between seeds of different developmental stages made it impossible to distinguish the three periods of Siberian wild rye based on LD1 and LD2 (**Figure 7A**). For multispectral features, the first two LDs accounted for 100% of the variance, and MRS seeds could be effectively separated in LD1 (**Figure 7B**). Similarly, for autofluorescence spectral features, the first two LDs explained 96.88% and 3.12% of the variance, respectively, but LD1 and LD2 were unable to separate seeds of the three periods of Siberian wild rye (**Figure 7C**). For multi-source fusion features, the first two LDs explained all of the variances, with LD1 and LD2 accounting for 92.16% and 7.84% of the variance, respectively. LD1 could completely distinguish MRS seeds, while LD2 could not separate DS and FRS seeds (**Figure 7D**). However, multi-source fused features could reveal more variation in seeds of Siberian wild rye at different maturity stages and could be employed as input features for the model.

### 3.3 Development of seed maturity models using multi-source fusion data and feature selection techniques

**Figure 8:**
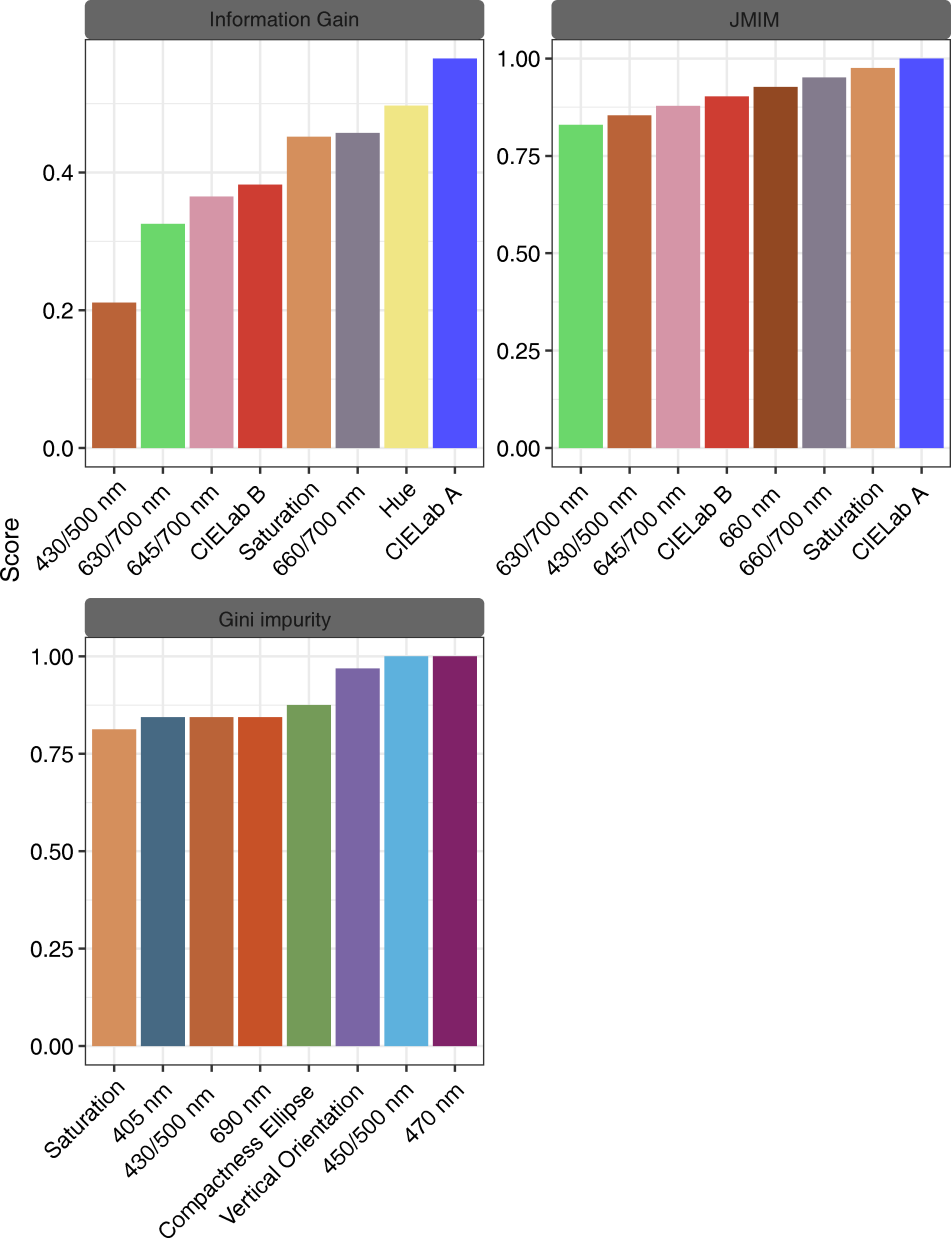
Feature importance scores of three feature filtering methods for information gain, JMIM, and Gini impurity.

We applied three feature filtering methods (JMIM, information gain, and Gini impurity) to calculate feature importance scores for all features. The top 20% of features were found to have higher scores, with the first 9 features for JMIM and Gini impurity and the first 8 features for Information Gain exceeding the respective scores (**Table S2**). Further analysis of the top 20% of features showed that the retained 8 features for Information Gain were primarily morphological and autofluorescence spectral features, with CIELab A, hue, 660/700 nm, saturation, and CIELab B being the top 5 features. The results from the JMIM method also showed that the first two most important features were morphological, with CIELab A being prominent among them. In addition, the multispectral feature 660 nm was also an important feature filtered by JMIM. In contrast, the top-scoring features of the Gini impurity method consisted mainly of multispectral features and autofluorescence features, and the CIELab A feature was not among the important features identified (**Figure 8**). Furthermore, the JMIM and information gain methods shared 5 common features, while the Gini impurity method had 6 exclusive features. In addition, the three methods shared 2 features, namely saturation and 430/500 nm (**Figure S1**).

We also evaluated their computational efficiency by measuring their running times. The results showed that the JMIM and information gain methods had shorter running times of 0.01 and 0.03 s, respectively. On the other hand, the Gini impurity method had the longest running time of 12.64 s (**Figure S2**). These results provide insight into the computational efficiency of the three feature filtering methods, which could help in selecting the appropriate method for a particular application.

**Figure 9:**
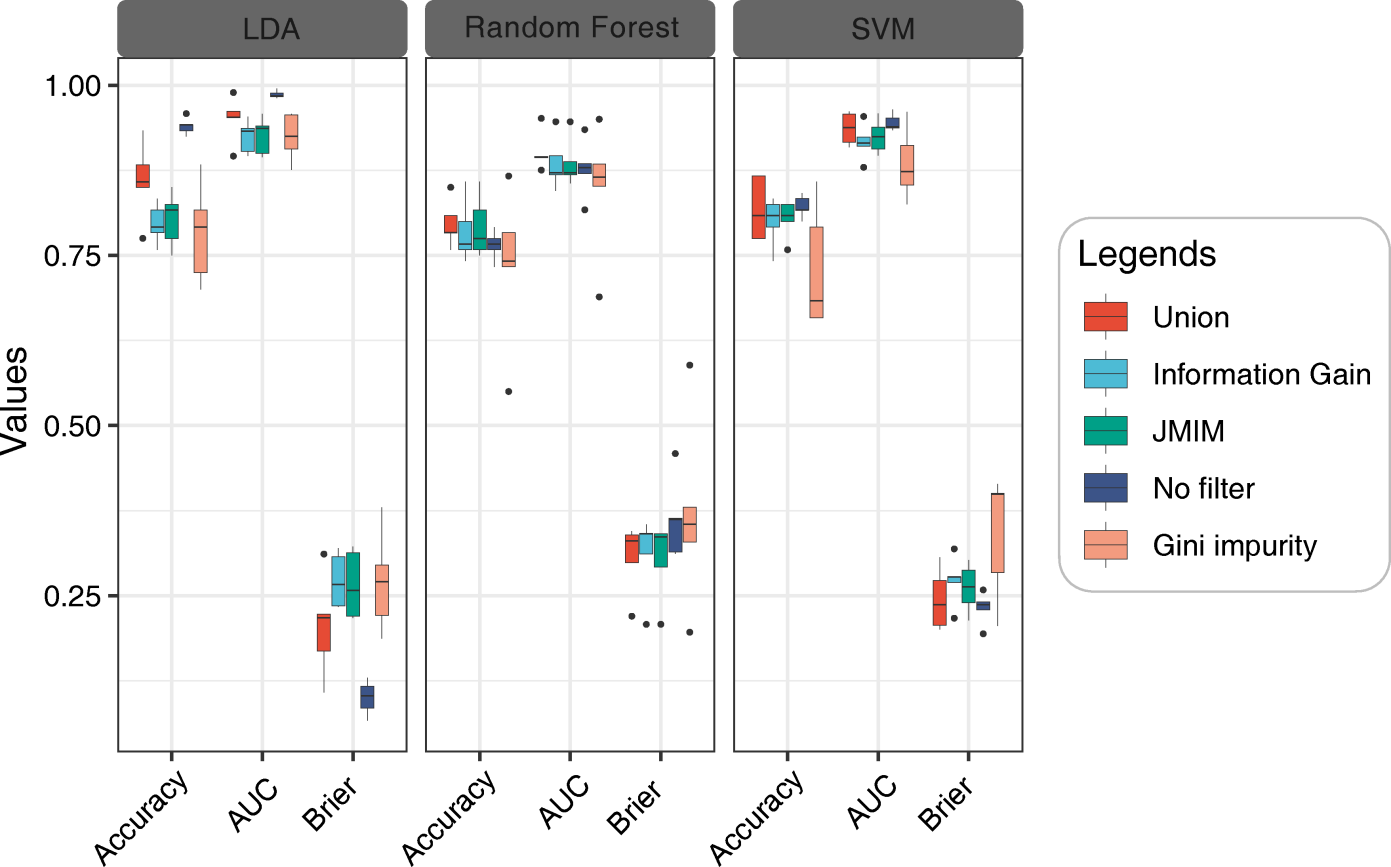
Model performance evaluation based on different feature filtering methods. From left to right are LDA, RF and SVM. Benchmark model performance with different filtering methods based on test set data using 5-fold cross-validation.

Further, we evaluated the performance of five datasets (three filtering algorithms, Union, and no-filtering) on LDA, RF, and SVM models (**Figure 9**). The results indicated that for the LDA model, the highest scores for accuracy, AUC, and Brier were achieved when the features were not filtered, with values of 0.94, 0.97, and 0.11, respectively. The feature fusion method was found to be the next best-performing method, while the remaining three methods demonstrated similar results. In the confusion matrix, MRS had the highest accuracy for seed identification, with an accuracy of approximately 1.00 under all five feature filtering strategies, while the DS had the lowest accuracy for seed detection (**Table S6**).

In the RF model, the feature fusion method had the best performance in terms of accuracy and AUC with values of 0.78 and 0.88, respectively, while the Gini impurity method had the lowest performance. However, the Brier scores were found to be similar across all five methods. The seed detection accuracy of MS was the highest, being greater than 0.96 in both the training and test sets, where the feature fusion-based method had the highest seed detection accuracy, followed by the JMIM method for the three stages (**Table S7**).

Lastly, for the SVM model, the AUC and Brier values were similar for both the feature fusion method and the no-filtering method, with the latter having the highest accuracy, while the Gini impurity method had the lowest value. The confusion matrix had the highest accuracy of seed detection without feature filtering, followed by the feature fusion method, while the JMIM and information gain methods had similar accuracies, both of which had about a 30% probability of identifying the DS seeds as FRS seeds (**Table S8**).

In summary, the results show that LDA consistently outperforms RF and SVM across all feature filtering methods, and we observe similar model performance using the feature fusion and JMIM methods, but JMIM has the shortest computation time.

**Figure 10:**
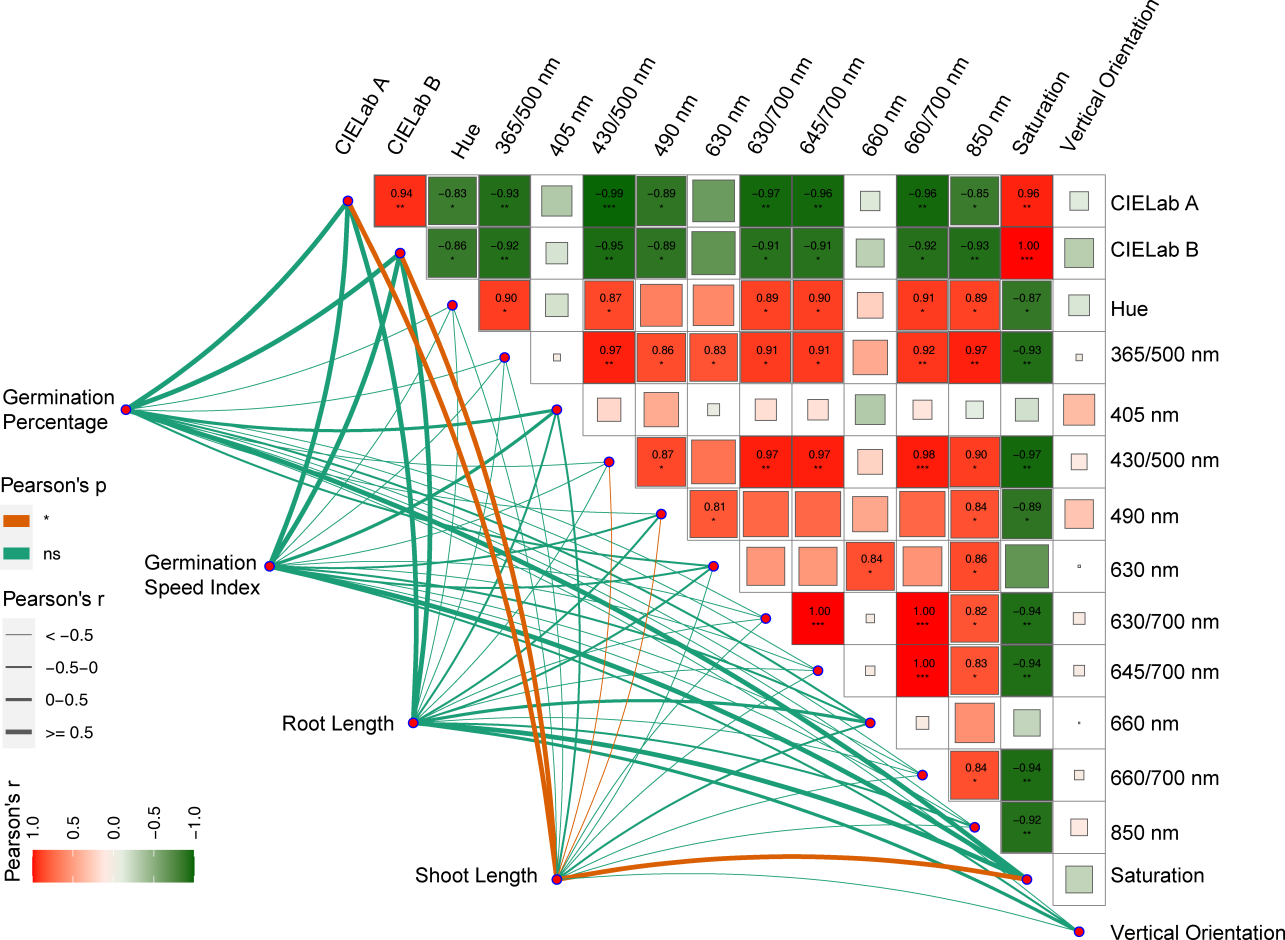
Pearson correlation coefficient of features based on the fusion of the three feature selection methods and Pearson correlation of germination indicators. The symbols '*', '**' and '***', indicate statistically significant correlation at P<0.05, P<0.01 and P<0.001 respectively, and ns indicates no correlation at P>0.05.

An analysis of Pearson’s correlations between 15 features, obtained by the fusion methods, and germination characteristics was performed (**Figure 10**). The results showed significant correlations (*P*<0.05) between the features, except 405 nm and vertical orientation. Notably, CIELab A and CIELab B exhibited a significant negative correlation with the remaining features, with the exception of saturation. Additionally, there was a significant positive correlation among the other features. Furthermore, it was found that shoot length was the only characteristic that was significantly correlated with the 15 features. Specifically, shoot length demonstrated a significant positive correlation with CIELab A, CIELab B, and saturation, and a significant negative correlation with 430/500 nm and 490 nm.

### 3.4 Validation and update of seed quality detection model based on multi-source fusion data

**Figure 11:**
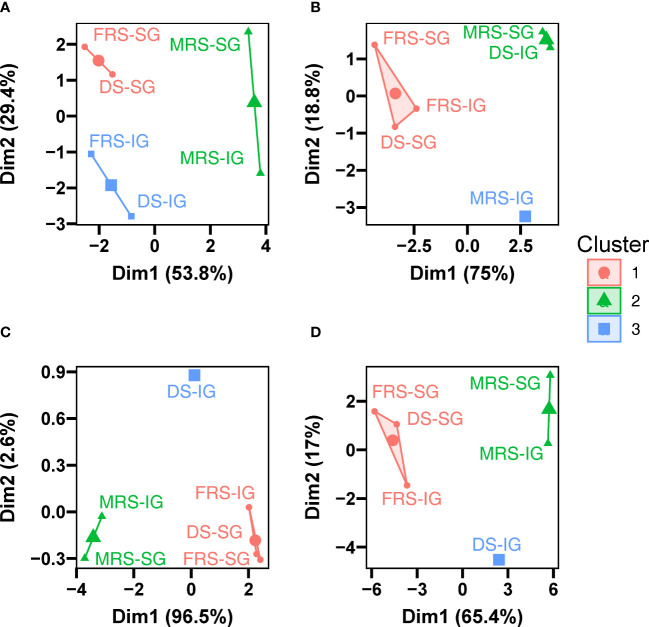
Two-dimensional biplot of seeds of different maturity stages and grain positions based on K-means clustering. **(A)** Morphological data, **(B)** multispectral data, **(C)** autofluorescence data, and **(D)** multi-source fusion data.

Due to the differences in SG and IG seeds at the same maturity stage, we applied k-means clustering techniques to improve model performance by reclassifying seeds at different stages and grain positions. The analysis showed that seeds of different maturity stages and grain positions were not grouped into one category based on maturity stage alone (**Figure 11**). Specifically, for the morphological data, seeds at different grain positions in the MRS were grouped into one class, while the IG seeds on the DS and FRS were grouped into another class, and the SG seeds on the FRS and DS were grouped into a third class. For the multispectral data, the IG seeds from the MRS were grouped in a separate class, the SG seeds from the MRS and the IG from the DS were grouped in another class, and the SG seeds from the DS and the SG and IG from the FRS were grouped in a third class. Similarly, for the autofluorescence data, the SG and IG seeds on the MRS group were grouped in one class, the IG seeds on the DS group in another class, and the SG seeds on the DS group and the SG and IG seeds on the FRS group in a third class. The results were consistent with the multispectral data when multi-source fusion data were used. These results suggest that describing the quality of Siberian wild rye seed on the basis of maturity stage alone may not be sufficient and that intra-stage variation needs to be considered.

**Figure 12:**
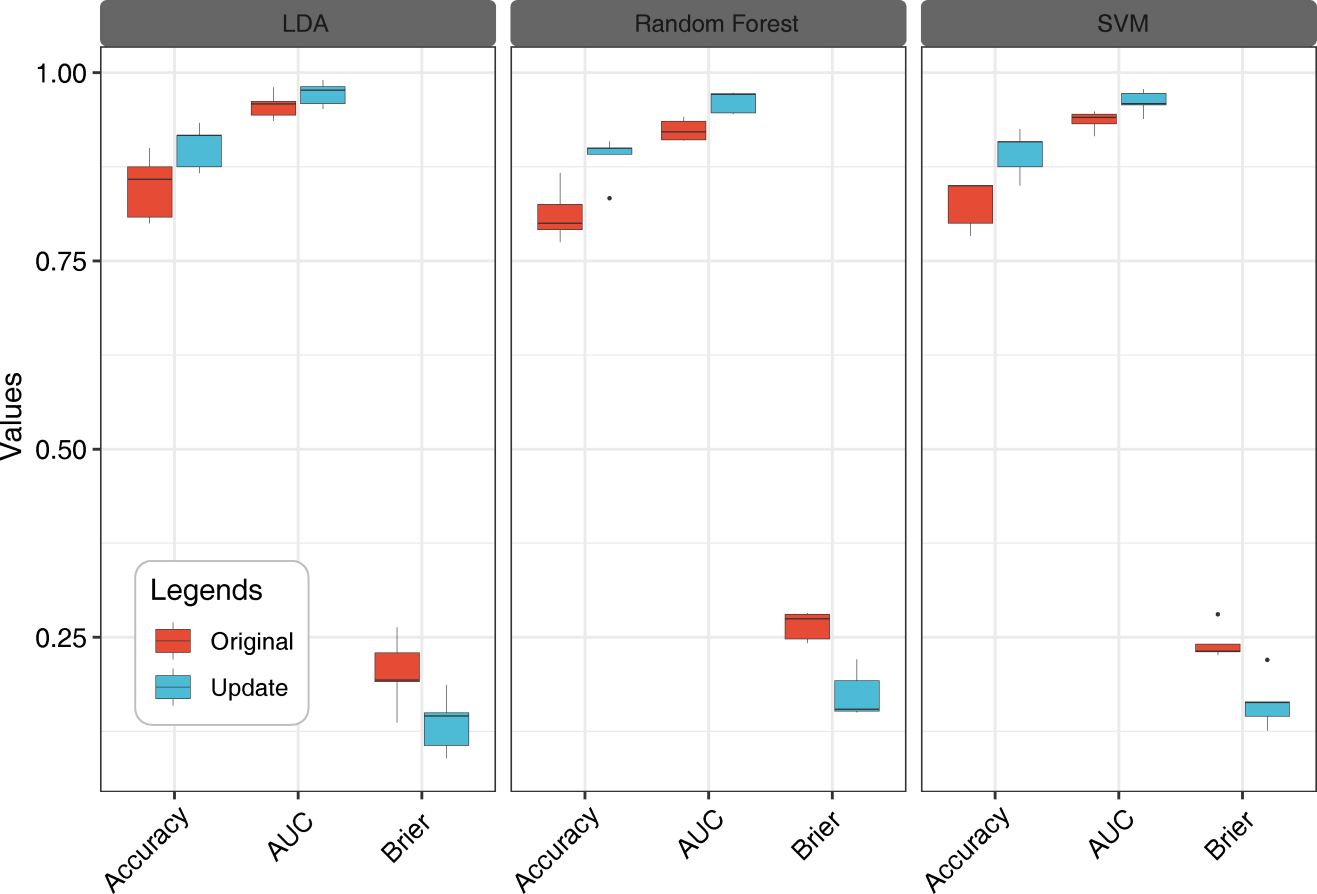
Accuracy of Siberian wild rye seed identification in three stages was improved by model update. Multi-source fusion data recognition models based on LDA, RF and SVM.

We employed K-means clustering based on feature fusion data, retaining only 20% of the original features, to improve the performance of the models. The results based on the test set showed a significant improvement in performance after reclassification (**Figure 12**). Specifically, the accuracy of the LDA model improved from 0.85 to 0.90, and the AUC improved from 0.95 to 0.97. In addition, the Brier score decreased from 0.20 to 0.14. Similarly, the accuracy of the RF model improved by 9.24% over the previous model to 0.87, and the AUC improved by 4.11%. The Brier score decreased from 0.27 to 0.20. The performance of the SVM model also improved, with the accuracy increasing from 0.83 to 0.89, and the AUC increasing by 2.63%. The Brier score decreased from 0.20 to 0.06.

In the confusion matrix, clust1 had the highest seed detection accuracy, followed by the detection accuracy of clust2, which was greater than 0.93 for all three models. Clust3 had the lowest detection accuracy, with the RF model having the lowest accuracy of 0.45 for its detection among the three models (**Table S9**).

Overall, these results suggest that label reclassification based on K-means clustering could substantially improve model performance for classifying seed maturity at different maturity stages and grain positions.

## 4 Discussion

Seed maturity is a crucial factor in improving the yield of Siberian wild rye, which is important for the sustainable development of animal husbandry and the improvement of degraded grasslands (Xie et al., 2015; Zhao et al., 2017). Delayed harvesting results in an 80% reduction in yield due to seed shattering (You et al., 2011), and traditionally, seed lots were sorted into different maturity fractions based on color, moisture content, and analysis of chlorophyll fluorescence signals (Jalink et al., 1998b; Ellis, 2019; Zhao et al., 2022). However, with advances in spectroscopy and computational technologies, non-destructive identification of seed characteristics is now possible through X-ray analysis (de Medeiros et al., 2020b), multispectral and hyperspectral image analysis (Xia et al., 2019), microtomography (Gomes-Junior et al., 2019), magnetic resonance (Melchinger et al., 2017), and other techniques. Recently, seed maturity analysis using multispectral imaging technology and ML methods has been applied to soybean (*Glycine max* L.) seed harvesting (Batista et al., 2022).

### 4.1 Seed maturity variation

Multispectral imaging has been demonstrated to be effective in differentiating seeds based on their morphological and spectral features (Hu et al., 2020). This study confirmed the morphological differences observed in previous reports during the maturation process of seeds (Zhang et al., 2022), where the projected area, length, and width of seeds increased with increasing maturity (Harada, 1997). However, these morphological traits were not sufficient to accurately determine seed maturity due to the differences between seeds with similar morphological features. The study showed that using saturation, CIELab A, and CIELab B probability density distribution trends, a combination of indicators that could considerably differentiate the maturity levels of Siberian wild rye seeds were identified. In addition, the study found that shoot length was positively correlated with CIELab A, CIELab B, and saturation, indicating its potential as an indicator to evaluate seed quality (Li and Chen, 2015).

Spectral information varies among species and varieties, as previous studies have shown (Zhang et al., 2012; Bao et al., 2019). In this study, we found that reflectance values were not uniform across seeds, although the spectral curves showed a similar trend. Immature seeds generally showed higher reflectance in the visible region of the spectrum due to changes in seed color and chlorophyll content (Boelt et al., 2018; ElMasry et al., 2019). The use of autofluorescence imaging was also found to be an effective tool for detecting fluorescent chemical compounds such as chlorophyll and lignin (Goggin and Steadman, 2012; Donaldson, 2020). Moreover, we found that excitation-emission combinations of 365/400 nm, 405/500 nm, 430/500 nm, 450/500 nm, and 660/700 nm provided clearer identification for three maturity stages seeds. This was consistent with previous researches (Jalink et al., 1998a; Kenanoglu et al., 2013; Donaldson and Williams, 2018). Furthermore, the use of supervised methods (LDA) was found to provide better results than unsupervised methods (PCA) in distinguishing seed maturity (Shrestha et al., 2016).

### 4.2 The feature filtering method could reduce the computational cost and time

In recent years, multispectral imaging designs have become more prevalent due to the shorter acquisition times required for image processing programs. This technology supports different LEDs light sources and allows for the precise stroboscopic timing needed to optimize and save images of each seed (ElMasry et al., 2019). Adjusting the light of each band individually can improve detection performance, making it crucial to select suitable features to improve training speed and reduce the operation cost of the model. While the SPA can reduce data dimension well in the hyperspectral spectrum, it may sacrifice accuracy as it relies on many linear relationships between the hyperspectral bands. Nonlinear relationships between self-fluorescence and morphological multivariate datasets require other methods to handle them. In this study, we used three filtering algorithms, we found that the feature filtering results of JMIM and information gain were similar in the first 20%, but different from Gini impurity as the latter relies on random forest models (Kursa, 2018; Zawadzki and Kosinski, 2019). Additionally, the running times of JMIM and information gain were substantially shorter than the Gini impurity, consistent with previous research (Bommert et al., 2020).

Feature filtering algorithms are increasingly used to improve ML models in the context of big data (Bommert et al., 2020). In this study, we applied three filtering algorithms to filter features, with no filtering and feature fusion as two comparison methods. Model performance was evaluated using these filtered features in LDA, RF, and SVM to automatically classify seed maturity. All models achieved accuracies greater than 0.78 and AUCs greater than 0.87, indicating that the classification process of Siberian wild rye seed maturity could be automated and provide reliable information on different maturity stages in a robust manner, similar to other studies (de Medeiros et al., 2020a; Barboza da Silva et al., 2021). Notably, feature filtering reduced the performance of the LDA model, while the unfiltered feature approach did not differ significantly from the other methods in RF and SVM. In addition, retaining the top 20% of features using filtering methods was found to be effective, but may result in a slight loss of accuracy. The feature fusion method outperformed other filtering methods such as information gain and Gini impurity, while the performance of JMIM was similar. The results obtained by combining different filtering methods could be used for key feature selection, and the optimal performance of the data training model was obtained under the JMIM filtering algorithm, which is consistent with previous research (Bommert et al., 2020).

### 4.3 The method based on K-means could improve the seed quality classification performance

The IG and SG seeds at the same maturity stage were varied reflecting on physiological, morphological, multispectral, and autofluorescence characteristics resulting in low model performance, this could be related to the low degree of domestication of Siberian wild rye. We re-classified 6 class seeds (3 stages×2 positions) into three clusters based on K-means. Interestingly, seeds at different grain positions varied in the same harvest time. IG matured later than SG about one week at the DS. Therefore, it may be inadequate to sort seed quality based on harvest time. Further, we found that the performance of the three models were improved by the model updating based on K-means clustering in Siberian wild rye seed quality classification. Moreover, this strategy could be employed to improve the performance of model recognition by applying it to other unknown maturity seeds not involved in model training. For instance, model performance was improved to identify other untested varieties by adding several untested corn varieties to the training data (Tu et al., 2022).

We have developed a fast, non-destructive, and high-throughput method to classify the maturity of Siberian wild rye seeds. This method could assist in determining the optimal harvest time in the field and is based on a feature filtering algorithm that can screen critical features, reducing equipment costs and training time. We could also develop low-cost instruments specifically for Siberian wild rye based on key features in the future. Compared to hyperspectral devices, our method was more cost-effective and flexible enough to train specific models for seed selection with unique features in different environments. Moreover, our feature filtering combined with machine learning algorithm could achieve optimal performance with smaller samples and shorter training time, unlike deep learning algorithms that require extensive data, parameter tuning, and training time.

While temperature, precipitation, and light affect seed maturity and phenotypic traits, our study showed the significant potential of the seed maturity classification model within a growth environment. We could use k-means clustering with standard samples to assign specific maturity labels for seeds in different environments to improve the model’s generalizability. In addition, future research could also enhance the accuracy and generalizability of this method by collecting seed samples from various growth environments.

## 5 Conclusion

In conclusion, our results demonstrated that integrated optical imaging technology has great potential for seed maturity identification in Siberian wild rye. The models based on multi-source fusion data showed wide applicability (>0.78 accuracies) and reduced the computing time and the cost of high-performance computing equipment. In addition, model updating based on K-means clustering could significantly improve model performance for seed maturity classification of plants with inconsistent maturity (low domestication).

## Data availability statement

The original contributions presented in the study are included in the article/**Supplementary Material**. Further inquiries can be directed to the corresponding author.

## Author contributions

PM conceived and designed the experiment. ZJ and CO performed the experiments and analyzed the data. SS, MS, JW, JL and WM contributed to the experiment. ZJ and CO wrote the paper, and SJ, ML, and PM revised the paper. All authors contributed to the article and approved the submitted version.

## Supplementary material

Supplementary Figure 1 Venn diagram based on information gain, Gini impurity, and JMIM feature filtering methods.

Supplementary Figure 2 Three feature filtering methods run time.
