# Supplementary figures and images for: Integrating optical imaging techniques for a novel approach to evaluate Siberian wild rye seed maturity
Source: Front Plant Sci. 2023 Apr 20;14:1170947. doi: 10.3389/fpls.2023.1170947 (PMC10157248; doi:10.3389/fpls.2023.1170947)

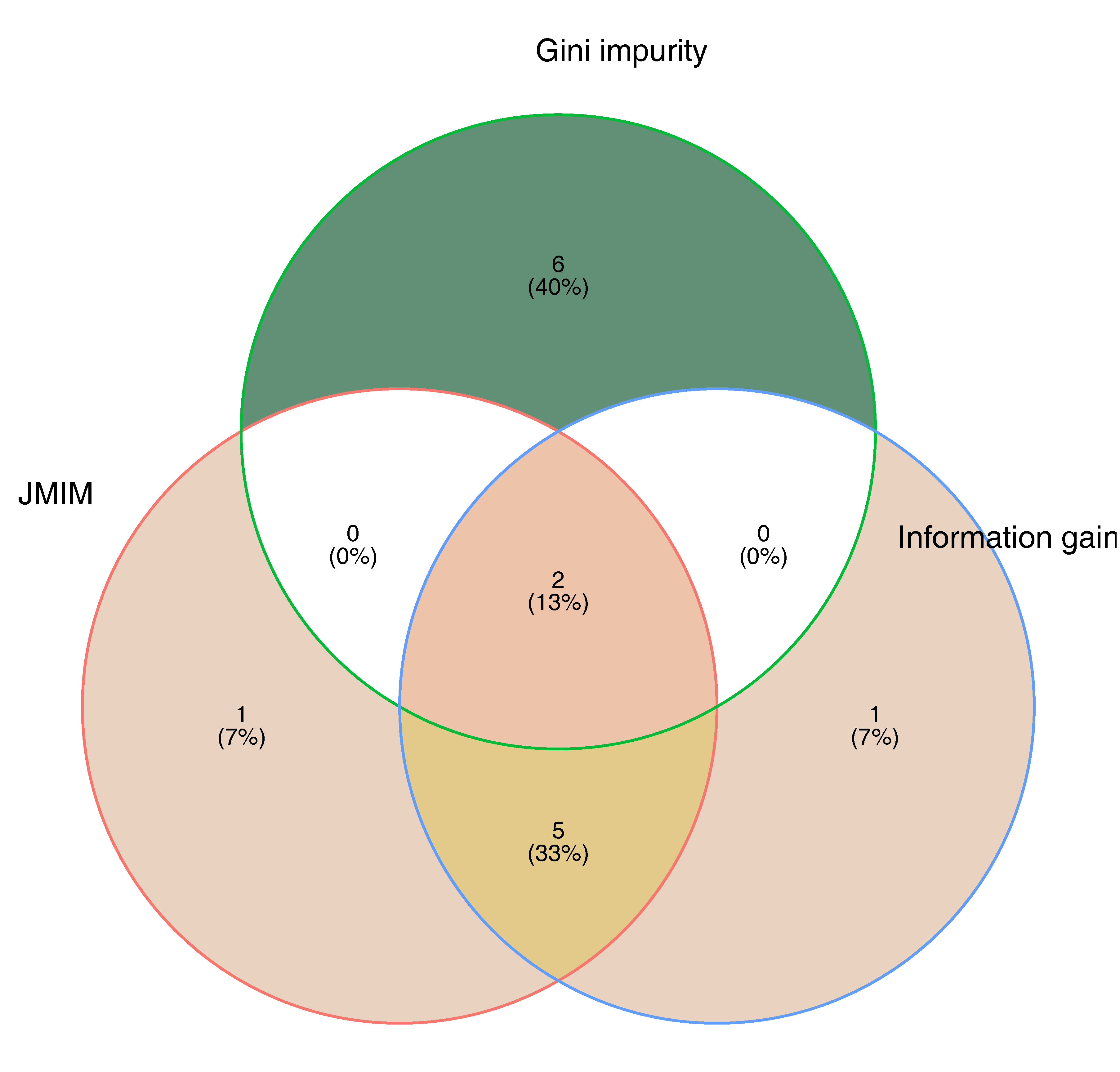

Supplement: Supplementary Figure 1 — Venn diagram based on information gain, Gini impurity, and JMIM feature filtering methods. [file Image_1.tif]

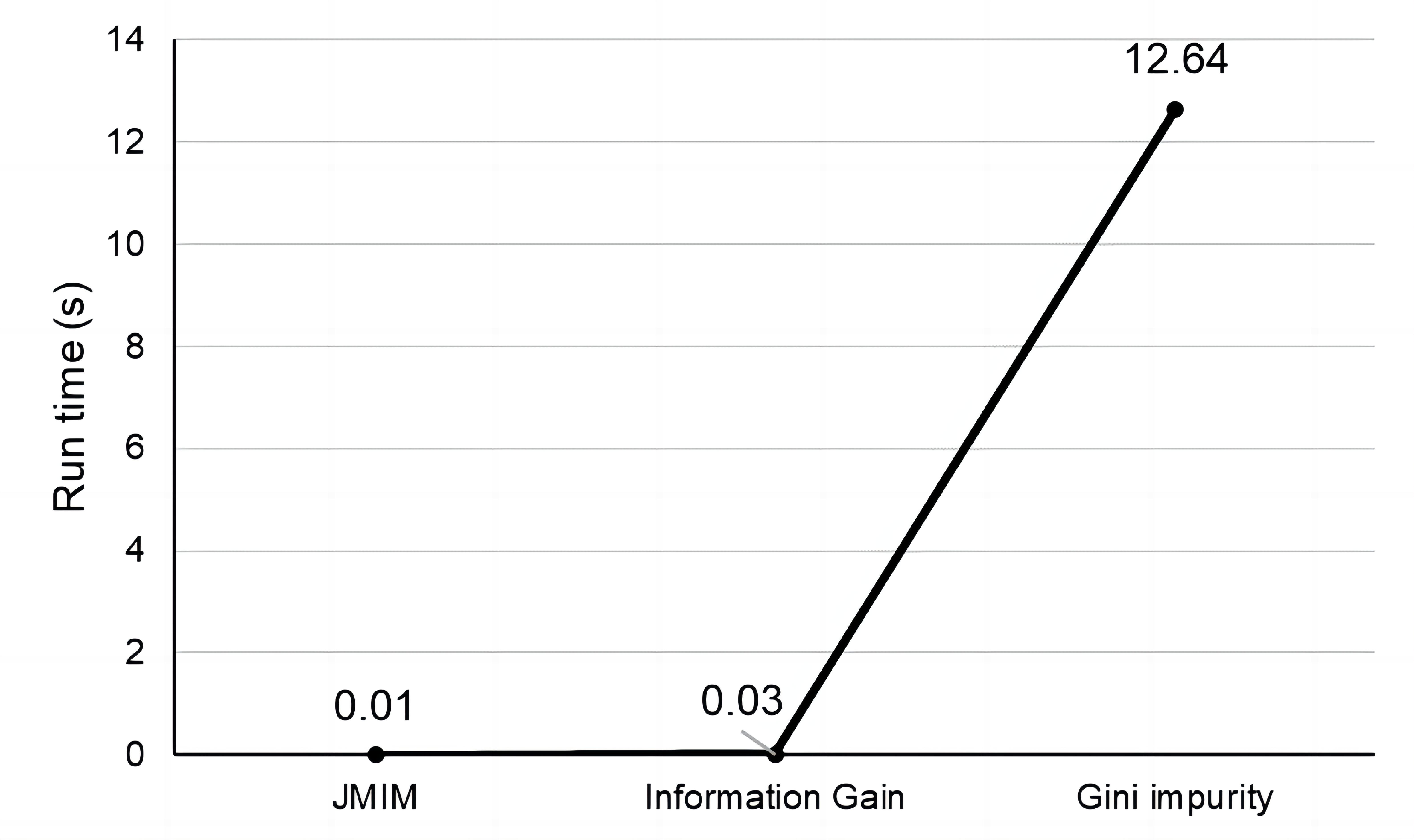

Supplement: Supplementary Figure 2 — Three feature filtering methods run time. [file Image_2.tif]
