# Supplementary material for: Integrating optical imaging techniques for a novel approach to evaluate Siberian wild rye seed maturity
Source: Front Plant Sci. 2023 Apr 20;14:1170947. doi: 10.3389/fpls.2023.1170947 (PMC10157248; doi:10.3389/fpls.2023.1170947)
Supplement: Supplementary file 3 [file Table_1.docx]

**Supplementary Table 1.** List of the extracted morphological variables from RGB images.

| Name | Feature | Description |
| --- | --- | --- |
| Area(mm^2^) | Binary Feature | Area of blob. |
| Length(mm) | Binary Feature | Length of blob bounding box. |
| Width(mm) | Binary Feature | Width of blob bounding box. |
| Width/Length Ratio | Shape Feature | Ratio of width to length of the image oriented bounding box. |
| Compactness Circle | Shape Feature | Compactness of blob defined as 4*Area/(π*length^2), ratio of object area to the area of a circle with the same  length (isolength quotient). |
| Compactness Ellipse | Shape feature | Compactness of blob defined as 4*Area/(π*length*width), ratio of object area to the area of an ellipse with the same length and width (isolength quotient). |
| BetaShape a | Shape Feature | Parameter a of beta-ellipse fitted to blob mask. Parameter a corresponds to width of most pointed blob-end. |
| BetaShape b | Shape Feature | Parameter b of beta-ellipse fitted to blob mask. Parameter b corresponds to width of least pointed blob-end. |
| Vertical orientation | Shape Feature | Skewness around the horizontal central axis. |
| Vertical skewness | Shape Feature | Vertical anisotropy index of characteristics. |
| CIELab L* | Color Feature | Mean Luminance component of CIELab-color of blob. |
| CIELab A* | Color Feature | Mean A-component of CIELab-color of blob. |
| CIELab B* | Color Feature | Mean B-component of CIELab-color of blob. |
| Saturation | Color Feature | Mean saturation of blob based on CIELab coordinates according to formulae: S = SQRT (A^2 + B^2). |
| Hue | Color Feature | Mean hue of blob based on CIELab coordinates according to formulae: H = ATAN (B/A). |
