# Supplementary material for: Integrating optical imaging techniques for a novel approach to evaluate Siberian wild rye seed maturity
Source: Front Plant Sci. 2023 Apr 20;14:1170947. doi: 10.3389/fpls.2023.1170947 (PMC10157248; doi:10.3389/fpls.2023.1170947)
Supplement: Supplementary file 4 [file Table_2.docx]

**Supplementary** **Table 2.** All 42 feature important scores based on three feature filtering algorithms.

| Feature | Score | Filter |
| --- | --- | --- |
| **CIELab A** | 1 | JMIM |
| **aturation** | 0.975609756 | JMIM |
| **660/700** | 0.951219512 | JMIM |
| **660** | 0.926829268 | JMIM |
| **CIELab B** | 0.902439024 | JMIM |
| **645/700** | 0.87804878 | JMIM |
| **430/500** | 0.853658537 | JMIM |
| **630/700** | 0.829268293 | JMIM |
| **780** | 0.804878049 | JMIM |
| 450 | 0.780487805 | JMIM |
| 405/500 | 0.756097561 | JMIM |
| CIELab L | 0.731707317 | JMIM |
| 365/400 | 0.707317073 | JMIM |
| Width | 0.682926829 | JMIM |
| 470 | 0.658536585 | JMIM |
| 450/500 | 0.634146341 | JMIM |
| 430 | 0.609756098 | JMIM |
| Betahape b | 0.585365854 | JMIM |
| 940 | 0.56097561 | JMIM |
| Ratio Width/Length | 0.536585366 | JMIM |
| Area | 0.512195122 | JMIM |
| Betahape a | 0.487804878 | JMIM |
| 540 | 0.463414634 | JMIM |
| 365 | 0.43902439 | JMIM |
| 405 | 0.414634146 | JMIM |
| Vertical Orientation | 0.390243902 | JMIM |
| Length | 0.365853659 | JMIM |
| 365/500nm | 0.341463415 | JMIM |
| Vertical kewne | 0.317073171 | JMIM |
| 490 | 0.292682927 | JMIM |
| Compactne Circle | 0.268292683 | JMIM |
| 850 | 0.243902439 | JMIM |
| 570 | 0.219512195 | JMIM |
| 590 | 0.195121951 | JMIM |
| Compactne Ellipe | 0.170731707 | JMIM |
| 515 | 0.146341463 | JMIM |
| 880 | 0.12195122 | JMIM |
| 970 | 0.097560976 | JMIM |
| Hue | 0.073170732 | JMIM |
| 690 | 0.048780488 | JMIM |
| 630 | 0.024390244 | JMIM |
| 645 | 0 | JMIM |
| **Betahape a** | 1 | Gini impurity |
| **CIELab A** | 1 | Gini impurity |
| **365** | 0.857142857 | Gini impurity |
| **CIELab B** | 0.642857143 | Gini impurity |
| **365/500nm** | 0.571428571 | Gini impurity |
| **880** | 0.571428571 | Gini impurity |
| **450/500nm** | 0.571428571 | Gini impurity |
| **CIELab L** | 0.571428571 | Gini impurity |
| **970** | 0.571428571 | Gini impurity |
| Betahape b | 0.357142857 | Gini impurity |
| 940 | 0.357142857 | Gini impurity |
| 590 | 0.285714286 | Gini impurity |
| 470 | 0.285714286 | Gini impurity |
| Hue | 0.285714286 | Gini impurity |
| 660 | 0.214285714 | Gini impurity |
| 405 | 0.214285714 | Gini impurity |
| Compactne Ellipe | 0.142857143 | Gini impurity |
| 430/500nm | 0.142857143 | Gini impurity |
| 850 | 0.142857143 | Gini impurity |
| 490 | 0.142857143 | Gini impurity |
| 780 | 0.142857143 | Gini impurity |
| 570 | 0.142857143 | Gini impurity |
| Width | 0.142857143 | Gini impurity |
| 365/400nm | 0.071428571 | Gini impurity |
| 430 | 0.071428571 | Gini impurity |
| 645/700nm | 0 | Gini impurity |
| Vertical Orientation | 0 | Gini impurity |
| 405/500nm | -0.071428571 | Gini impurity |
| 450 | -0.071428571 | Gini impurity |
| 515 | -0.142857143 | Gini impurity |
| aturation | -0.214285714 | Gini impurity |
| Area | -0.214285714 | Gini impurity |
| Length | -0.285714286 | Gini impurity |
| 690 | -0.285714286 | Gini impurity |
| Vertical kewne | -0.357142857 | Gini impurity |
| 660/700nm | -0.428571429 | Gini impurity |
| 540 nm | -0.428571429 | Gini impurity |
| 630 | -0.428571429 | Gini impurity |
| Compactne Circle | -0.5 | Gini impurity |
| 630/700nm | -0.5 | Gini impurity |
| 645 | -0.642857143 | Gini impurity |
| Ratio Width/Length | -1.285714286 | Gini impurity |
| **CIELab A** | 0.565548086 | Information Gain |
| **Hue** | 0.49711113 | Information Gain |
| **660/700nm** | 0.457238595 | Information Gain |
| **aturation** | 0.451852918 | Information Gain |
| **CIELab B** | 0.382203038 | Information Gain |
| **645/700nm** | 0.36520595 | Information Gain |
| **630/700nm** | 0.325166597 | Information Gain |
| 430/500nm | 0.21127316 | Information Gain |
| 405/500nm | 0.170731176 | Information Gain |
| 365/400nm | 0.167037342 | Information Gain |
| 450 | 0.136021109 | Information Gain |
| Width | 0.127726178 | Information Gain |
| 450/500nm | 0.123453077 | Information Gain |
| 470 | 0.110726437 | Information Gain |
| CIELab L | 0.10391052 | Information Gain |
| 540 nm | 0.082338947 | Information Gain |
| 780 | 0.078563319 | Information Gain |
| 430 | 0.07677057 | Information Gain |
| Ratio Width/Length | 0.065637603 | Information Gain |
| 490 | 0.063592852 | Information Gain |
| 365/500nm | 0.061275594 | Information Gain |
| 880 | 0.057949844 | Information Gain |
| Area | 0.055510966 | Information Gain |
| 850 | 0.052535934 | Information Gain |
| Betahape b | 0.051454907 | Information Gain |
| 515 | 0.049935241 | Information Gain |
| Betahape a | 0.046545481 | Information Gain |
| 570 | 0.043052887 | Information Gain |
| 940 | 0.036875047 | Information Gain |
| Compactne Circle | 0.035341924 | Information Gain |
| 970 | 0.034173846 | Information Gain |
| 405 | 0 | Information Gain |
| Length | 0 | Information Gain |
| 630 | 0 | Information Gain |
| 690 | 0 | Information Gain |
| 365 | 0 | Information Gain |
| 660 | 0 | Information Gain |
| Compactne Ellipe | 0 | Information Gain |
| Vertical kewne | 0 | Information Gain |
| 590 | 0 | Information Gain |
| 645 | 0 | Information Gain |
| Vertical Orientation | 0 | Information Gain |
