# Supplementary material for: Integrating optical imaging techniques for a novel approach to evaluate Siberian wild rye seed maturity
Source: Front Plant Sci. 2023 Apr 20;14:1170947. doi: 10.3389/fpls.2023.1170947 (PMC10157248; doi:10.3389/fpls.2023.1170947)
Supplement: Supplementary file 5 [file Table_3.docx]

| **Supplementary** **Table 3.** Random-research details of partial hyperparameters of the three models. | | | |
| --- | --- | --- | --- |
| Models | Filter methods | Optimization Range of Partial Hyperparameters | Tuned Hyperparameters |
| RF | JMIM | ntree = [1:500]; mtry = [1:500]; nodesize = [1:50] | ntree = 303;mtry = 185; nodesize = 34 |
|  | PI | ntree = [1:500]; mtry = [1:500]; nodesize = [1:51] | ntree = 499;mtry = 308; nodesize = 4 |
|  | ING | ntree = [1:500]; mtry = [1:500]; nodesize = [1:52] | ntree = 17;mtry = 54; nodesize = 21 |
| SVM | JMIM | cost = [0:2]; gamma = [0:2]; kernel = ['polynomial','radial','sigmoid'] | cost = 0.48; gamma = 1.12; kernel = 'radial' |
|  | PI | cost = [0:2];gamma = [0:3]; kernel = ['polynomial','radial','sigmoid'] | cost = 0.79; gamma = 0.21; kernel = 'radial' |
|  | ING | cost = [0:2]; gamma = [0:4]; kernel = ['polynomial','radial','sigmoid'] | cost = 0.24; gamma = 0.37; kernel = 'radial' |
