# Supplementary material for: Integrating optical imaging techniques for a novel approach to evaluate Siberian wild rye seed maturity
Source: Front Plant Sci. 2023 Apr 20;14:1170947. doi: 10.3389/fpls.2023.1170947 (PMC10157248; doi:10.3389/fpls.2023.1170947)
Supplement: Supplementary file 6 [file Table_4.docx]

**Supplementary Table 4.** Description of morphological features of Siberian wild rye seeds at different maturity stages and grain positions.

| Features | MRS-IG | MRS-SG | DS-IG | DS-SG | FRS-IG | FRS-SG |
| --- | --- | --- | --- | --- | --- | --- |
| Area | 11.18±1.4e | 13.11±1.42c | 11.62±1.71d | 13.66±1.4b | 12.74±1.69c | 14.27±1.53a |
| BetaShape a | 1.81±0.2b | 1.86±0.19b | 1.98±0.24a | 1.95±0.21a | 1.96±0.19a | 1.95±0.19a |
| BetaShape b | 1.65±0.14b | 1.63±0.12b | 1.77±0.16a | 1.73±0.15a | 1.75±0.14a | 1.73±0.13a |
| CIELab A | 1.39±0.69e | 1.68±0.58d | 3.96±0.98c | 4.97±1.23b | 4.79±0.96b | 5.6±1.05a |
| CIELab B | 14.53±1.1e | 13.5±1.32f | 15.96±2.12d | 17.09±2.44c | 18.21±1.44b | 19.07±1.53a |
| CIELab L | 54.95±1.41a | 53.66±1.68b | 53.67±1.95b | 51.67±2.36d | 52.82±1.9c | 51.79±1.71d |
| Compactness Circle | 0.16±0.02c | 0.15±0.01d | 0.17±0.02a | 0.16±0.01c | 0.16±0.01b | 0.15±0.01c |
| Compactness Ellipse | 0.99±0.01a | 0.98±0.01a | 0.98±0.01a | 0.98±0.01a | 0.98±0.01a | 0.98±0.01a |
| Hue | 1.35±0.59b | 1.44±0.06a | 1.32±0.06b | 1.28±0.05b | 1.31±0.04b | 1.28±0.04b |
| Length | 9.68±0.81d | 10.77±0.82b | 9.62±0.95d | 10.78±0.71b | 10.21±0.92c | 11.09±0.87a |
| RatioWidth/Length | 0.17±0.02b | 0.16±0.01c | 0.18±0.02a | 0.17±0.01b | 0.18±0.01a | 0.17±0.01b |
| Saturation | 14.28±1.13e | 13.27±1.3f | 16.5±2.33d | 17.93±2.68c | 18.95±1.58b | 19.99±1.65a |
| Vertical Orientation | 0.06±0.08ab | 0.04±0.07bc | 0.06±0.09ab | 0.08±0.07a | 0.03±0.07c | 0.04±0.06c |
| Vertical Skewness | -0.07±0.06a | -0.1±0.05c | -0.08±0.05ab | -0.09±0.04bc | -0.09±0.05ab | -0.09±0.04bc |
| Width | 1.62±0.13d | 1.72±0.14c | 1.75±0.14c | 1.84±0.12ab | 1.82±0.13b | 1.87±0.1a |
