# Supplementary material for: Integrating optical imaging techniques for a novel approach to evaluate Siberian wild rye seed maturity
Source: Front Plant Sci. 2023 Apr 20;14:1170947. doi: 10.3389/fpls.2023.1170947 (PMC10157248; doi:10.3389/fpls.2023.1170947)
Supplement: Supplementary file 7 [file Table_5.docx]

**Supplementary Table 5.** Description of multispectral features of Siberian wild rye seeds at different maturity stages and grain positions.

| Wavelengths (nm) | DS_IG | DS_SG | FRS_IG | FRS_SG | MRS_IG | MRS_SG |
| --- | --- | --- | --- | --- | --- | --- |
| 365 | 11.3±1.3b | 11.67±1.1a | 11.67±1.06a | 11.32±1.17b | 11.99±1.07a | 10.57±1.11c |
| 405 | 13.36±1.47bc | 13.58±1.2b | 13.58±1.17b | 13.13±1.32c | 14.08±1.14a | 13.1±1.35c |
| 430 | 14.94±1.34b | 14.69±1.17b | 14.59±1.12b | 14.07±1.27c | 15.74±0.95a | 14.87±1.05b |
| 450 | 15.82±1.11b | 15.34±1.12c | 15.26±1.07c | 14.76±1.16d | 16.36±0.95a | 15.85±0.85b |
| 470 | 15.58±1.06b | 15.06±1.11c | 15.04±1.03c | 14.6±1.12d | 15.95±1.02a | 15.57±0.93b |
| 490 | 15±1.08a | 14.46±1.11b | 14.5±1.01b | 14.14±1.09c | 15.2±1.09a | 14.96±1.03a |
| 515 | 14.62±1.37a | 13.83±1.24b | 13.96±1.09b | 13.63±1.15b | 14.77±1.41a | 14.68±1.52a |
| 540 | 15.65±1.49a | 14.7±1.38bc | 14.85±1.19b | 14.43±1.24c | 16.02±1.53a | 15.74±1.68a |
| 570 | 14.58±1.85a | 13.64±1.42b | 13.8±1.24b | 13.56±1.29b | 14.72±1.81a | 14.51±2.09a |
| 590 | 14.6±1.98a | 13.6±1.47b | 13.87±1.26b | 13.69±1.33b | 14.44±1.86a | 14.39±2.18a |
| 630 | 15.43±2.26a | 14.28±1.61d | 14.62±1.39bcd | 14.47±1.45cd | 15±2.05abc | 15.15±2.44ab |
| 645 | 16.33±2.47a | 15.03±1.76d | 15.39±1.57bcd | 15.2±1.6cd | 15.75±2.19abc | 15.91±2.63ab |
| 660 | 16.39±2.55a | 15.12±1.79b | 15.4±1.52b | 15.3±1.59b | 15.28±2.1b | 15.65±2.54b |
| 690 | 17.48±2.92a | 15.94±2c | 16.18±1.7c | 16.06±1.74c | 16.4±2.54bc | 17.03±3.15ab |
| 780 | 21.17±3.9b | 18.96±2.55c | 19.03±2.11c | 18.97±2.15c | 21.34±4.34b | 23.05±5.81a |
| 850 | 32.52±3.51b | 30.38±2.3c | 30.41±1.91c | 30.18±1.87c | 32.21±3.76b | 33.71±5.16a |
| 880 | 37.31±3.22ab | 35.29±2.15c | 35.32±1.81c | 35.02±1.75c | 36.89±3.42b | 38.08±4.72a |
| 940 | 47.58±2.65a | 45.81±1.84c | 45.93±1.61c | 45.54±1.57c | 46.66±2.72b | 47.51±3.86a |
| 970 | 50.66±2.5a | 48.93±1.76bc | 49.11±1.56bc | 48.7±1.53c | 49.51±2.54b | 50.31±3.65a |
