# Supplementary material for: Integrating optical imaging techniques for a novel approach to evaluate Siberian wild rye seed maturity
Source: Front Plant Sci. 2023 Apr 20;14:1170947. doi: 10.3389/fpls.2023.1170947 (PMC10157248; doi:10.3389/fpls.2023.1170947)
Supplement: Supplementary file 8 [file Table_6.docx]

**Supplementary Table 6.** Confusion matrix based on five filtering methods of LDA model.

|  |  | Train(n=160) | | | | Test(n=40) | |  |  |
| --- | --- | --- | --- | --- | --- | --- | --- | --- | --- |
|  | Prediction | Reference | | | | | | | |
|  |  |  |  |  | Total |  |  |  | Total |
|  |  | FRS | DS | MRS |  | FRS | DS | MRS |  |
| JMIM | FRS | 117 | 41 | 0 | - | 32 | 13 | 0 | - |
|  | DS | 43 | 114 | 0 | - | 8 | 25 | 0 | - |
|  | MRS | 0 | 5 | 160 | - | 0 | 2 | 40 | - |
|  | Accuracy | 0.74 | 0.55 | 1 | 0.81 | 0.80 | 0.63 | 1 | 0.81 |
| Gini impurity | FRS | 121 | 45 | 0 | - | 28 | 15 | 0 | - |
|  | DS | 39 | 113 | 3 | - | 10 | 24 | 0 | - |
|  | MRS | 0 | 2 | 157 | - | 2 | 1 | 40 | - |
|  | Accuracy | 0.65 | 0.55 | 1 | 0.81 | 0.7 | 0.6 | 1 | 0.77 |
| Information Gain | FRS | 116 | 43 | 0 | - | 33 | 13 | 0 | - |
|  | DS | 44 | 112 | 1 | - | 7 | 24 | 1 | - |
|  | MRS | 0 | 5 | 159 | - | 0 | 3 | 39 | - |
|  | Accuracy | 0.68 | 0.64 | 0.99 | 0.81 | 0.8 | 0.60 | 0.99 | 0.80 |
| Union | FRS | 126 | 39 | 0 | - | 33 | 9 | 0 | - |
|  | DS | 34 | 119 | 0 | - | 7 | 31 | 0 | - |
|  | MRS | 0 | 2 | 160 | - | 0 | 0 | 40 | - |
|  | Accuracy | 0.75 | 0.72 | 1 | **0.87** | 0.95 | 0.8 | 1 | **0.87** |
| No_filtering | FRS | 156 | 13 | 0 | - | 39 | 6 | 0 | - |
|  | DS | 4 | 147 | 1 | - | 1 | 34 | 0 | - |
|  | MRS | 0 | 0 | 159 | - | 0 | 0 | 40 | - |
|  | Accuracy | 0.87 | 0.82 | 1 | **0.96** | 0.98 | 0.85 | 1 | **0.94** |
