# Supplementary material for: Integrating optical imaging techniques for a novel approach to evaluate Siberian wild rye seed maturity
Source: Front Plant Sci. 2023 Apr 20;14:1170947. doi: 10.3389/fpls.2023.1170947 (PMC10157248; doi:10.3389/fpls.2023.1170947)
Supplement: Supplementary file 9 [file Table_7.docx]

|  |  | Train(n=160) | | | | Test(n=40) | |  |  |
| --- | --- | --- | --- | --- | --- | --- | --- | --- | --- |
|  | Prediction | Reference | | | | | | | |
|  |  |  |  |  | Total |  |  |  | Total |
|  |  | FRS | DS | MRS |  | FRS | DS | MRS |  |
| JMIM | FRS | 131 | 49 | 0 | - | 33 | 15 | 0 | - |
|  | DS | 27 | 108 | 1 | - | 7 | 24 | 1 | - |
|  | MRS | 2 | 3 | 159 | - | 0 | 1 | 39 | - |
|  | Accuracy | 0.82 | 0.68 | 0.99 | 0.83 | 0.83 | 0.6 | 0.98 | 0.77 |
| Gini impurity | FRS | 130 | 48 | 0 | - | 30 | 14 | 0 | - |
|  | DS | 29 | 105 | 6 | - | 9 | 24 | 1 | - |
|  | MRS | 1 | 7 | 154 | - | 1 | 2 | 39 | - |
|  | Accuracy | 0.81 | 0.66 | 0.96 | 0.81 | 0.75 | 0.6 | 0.98 | 0.73 |
| Information Gain | FRS | 132 | 60 | 0 | - | 30 | 14 | 0 | - |
|  | DS | 28 | 99 | 2 | - | 10 | 26 | 1 | - |
|  | MRS | 0 | 1 | 158 | - | 0 | 0 | 39 | - |
|  | Accuracy | 0.83 | 0.62 | 0.99 | 0.81 | 0.75 | 0.65 | 0.98 | 0.76 |
| Union | FRS | 138 | 49 | 0 | - | 31 | 13 | 0 | - |
|  | DS | 21 | 107 | 1 | - | 9 | 29 | 0 | - |
|  | MRS | 0 | 4 | 159 | - | 0 | 0 | 40 | - |
|  | Accuracy | 0.86 | 0.67 | 0.99 | **0.84** | 0.78 | 0.73 | 1 | **0.78** |
| No_filtering | FRS | 129 | 45 | 0 | - | 27 | 14 | 0 | - |
|  | DS | 31 | 114 | 5 | - | 20 | 24 | 5 | - |
|  | MRS | 0 | 1 | 155 | - | 3 | 2 | 39 | - |
|  | Accuracy | 0.81 | 0.71 | 0.97 | 0.83 | 0.67 | 0.6 | 0.98 | 0.75 |

**Supplementary Table 7.** Confusion matrix based on five filtering methods of RF model.
