# Supplementary material for: Integrating optical imaging techniques for a novel approach to evaluate Siberian wild rye seed maturity
Source: Front Plant Sci. 2023 Apr 20;14:1170947. doi: 10.3389/fpls.2023.1170947 (PMC10157248; doi:10.3389/fpls.2023.1170947)
Supplement: Supplementary file 10 [file Table_8.docx]

**Supplementary Table 8.** Confusion matrix based on five filtering methods of SVM model.

|  |  | Train(n=160) | | | | Test(n=40) | |  |  |
| --- | --- | --- | --- | --- | --- | --- | --- | --- | --- |
|  | Prediction | Reference | | | | | | | |
|  |  |  |  |  | Total |  |  |  | Total |
|  |  | FRS | DS | MRS |  | FRS | DS | MRS |  |
| JMIM | FRS | 139 | 45 | 0 | - | 32 | 12 | 0 | - |
|  | DS | 21 | 114 | 1 | - | 8 | 28 | 1 | - |
|  | MRS | 0 | 1 | 159 | - | 0 | 0 | 39 | - |
|  | Accuracy | 0.87 | 0.71 | 0.99 | 0.86 | 0.8 | 0.7 | 0.98 | 0.83 |
| Gini impurity | FRS | 131 | 43 | 0 | - | 30 | 14 | 0 | - |
|  | DS | 28 | 112 | 4 | - | 9 | 24 | 1 | - |
|  | MRS | 1 | 5 | 156 | - | 1 | 2 | 39 | - |
|  | Accuracy | 0.82 | 0.7 | 0.98 | 0.83 | 0.75 | 0.6 | 0.98 | 0.78 |
| Information Gain | FRS | 132 | 60 | 0 | - | 30 | 14 | 0 | - |
|  | DS | 28 | 99 | 2 | - | 10 | 26 | 1 | - |
|  | MRS | 0 | 1 | 158 | - | 0 | 0 | 39 | - |
|  | Accuracy | 0.83 | 0.62 | 0.99 | 0.81 | 0.75 | 0.65 | 0.98 | 0.79 |
| Union | FRS | 138 | 39 | 0 | - | 31 | 13 | 0 | - |
|  | DS | 21 | 120 | 1 | - | 9 | 29 | 0 | - |
|  | MRS | 0 | 1 | 159 | - | 0 | 0 | 40 | - |
|  | Accuracy | 0.86 | 0.75 | 0.99 | **0.87** | 0.78 | 0.73 | 1 | **0.84** |
| No_filtering | FRS | 139 | 23 | 0 | - | 33 | 12 | 0 | - |
|  | DS | 21 | 136 | 0 | - | 7 | 29 | 0 | - |
|  | MRS | 0 | 1 | 160 | - | 0 | 0 | 40 | - |
|  | Accuracy | 0.87 | 0.85 | 1 | 0.91 | 0.83 | 0.73 | 1 | 0.85 |
