# Supplementary material for: Integrating optical imaging techniques for a novel approach to evaluate Siberian wild rye seed maturity
Source: Front Plant Sci. 2023 Apr 20;14:1170947. doi: 10.3389/fpls.2023.1170947 (PMC10157248; doi:10.3389/fpls.2023.1170947)
Supplement: Supplementary file 11 [file Table_9.docx]

**Supplementary Table 9.** Confusion matrix of LDA, RF and SVM models after updating the model based on K-means

|  |  | Train |  |  |  | Test |  |  |  |
| --- | --- | --- | --- | --- | --- | --- | --- | --- | --- |
|  | Prediction | Refrence | | | | | | | |
| Models |  | clust1 | clust2 | clust3 | Total | clust1 | clust2 | clust3 | Total |
| LDA | clust1 | 160 | 0 | 1 | - | 40 | 0 | 1 | - |
|  | clust2 | 0 | 222 | 22 | - | 0 | 52 | 7 | - |
|  | clust3 | 0 | 18 | 57 | - | 0 | 8 | 12 | - |
|  | Accuracy | 1 | 0.93 | 0.71 | 0.91 | 1 | 0.87 | 0.6 | 0.90 |
| RF | clust1 | 160 | 0 | 0 | - | 40 | 0 | 2 | - |
|  | clust2 | 0 | 240 | 0 | - | 0 | 55 | 9 | - |
|  | clust3 | 0 | 0 | 80 | - | 0 | 5 | 9 | - |
|  | Accuracy | 1 | 1 | 1 | 1 | 1 | 0.92 | 0.45 | 0.87 |
| SVM | clust1 | 158 | 0 | 1 | - | 40 | 0 | 0 | - |
|  | clust2 | 0 | 232 | 25 | - | 0 | 55 | 9 | - |
|  | clust3 | 2 | 8 | 54 | - | 0 | 5 | 11 | - |
|  | Accuracy | 0.99 | 0.97 | 0.68 | 0.93 | 1 | 0.92 | 0.55 | 0.89 |

Note: Number of clust1, clust2 and clust3 were 200, 300 and 100, respectively.
